# Gynecomastia and drugs: a critical evaluation of the literature

**DOI:** 10.1007/s00228-015-1835-x

**Published:** 2015-04-02

**Authors:** Frank Q. Nuttall, Rohit S. Warrier, Mary C. Gannon

**Affiliations:** 1 Section of Endocrinology, Metabolism and Nutrition, Minneapolis VA Health Care System, One VeteransDrive, Minneapolis, MN 55417 USA; 2 The Department of Medicine, Academic Health Center, University of Minnesota, Minneapolis, MN 55455 USA; 3 The Department of Food Science and Nutrition, University of Minnesota, St. Paul, MN 55108 USA

**Keywords:** Spironolactone, Mastodynia, Gynecomastia, Andromastia

## Abstract

**Purpose:**

A large number of medications have been implicated in the genesis of gynecomastia. However, gynecomastia is common in men, asymptomatic, increases with age, and is considered to be due to an increased estradiol/testosterone ratio. This complicates the interpretation of medication-related gynecomastia. Therefore, we have reviewed the literature in order to assess the data relating gynecomastia onset with utilization of specific medications.

**Methods:**

The literature was searched in PubMed and the Ovid/Medline databases from the 1946 to January 2015 with the search terminology of “gynecomastia, drugs/medications.” A few other articles were found and included.

**Results:**

One hundred ten publications were reviewed. Sixty-three were single case reports. There were 24 population-based studies of which 8 were HIV-infected patients treated with antiretroviral agents. Among the case reports, 49 were for individual medications, and 2 were reports of antineoplastic or antiretroviral drug regimens. In the great majority, mastodynia with or without breast enlargement was present and referred to as gynecomastia. Generally, hormonal profiles could not explain the breast enlargement. The pain/tenderness and breast enlargement resolved spontaneously over time.

**Conclusion:**

Many different medications have been associated with the presence of “gynecomastia.” Generally, it presents as a syndrome characterized by a single painful/tender breast (mastodynia) associated with breast enlargement and is transient. We suggest that these cases be referred to as an acute gynecomastia syndrome. This syndrome also occurs independent of medication use. Thus, in an individual patient, whether it is medication induced often remains uncertain. The pathogenesis remains unknown.

## Introduction

Gynecomastia, as currently defined, represents the presence of palpable, subareolar breast tissue with a diameter of 2 cm or greater. Generally, it is asymptomatic, not noted by the patients, and the prevalence increases with aging and obesity [1]. The etiology is uncertain but has been attributed to an increase in the estradiol/testosterone ratio [2, 3].

Numerous medications have been reported to be associated with the development of gynecomastia. The implication is that these drugs were responsible for its appearance or progression and this is due to a change in the sex hormone status. In this review, we have systematically searched the literature regarding drugs suspected to be etiologically related to the clinical manifestation of breast enlargement, i.e., the development of gynecomastia. Drug implications are particularly important because it has been clearly shown that gynecomastia, as defined above is asymptomatic, and a common finding in males. Indeed, the prevalence has been reported to be 36 % in younger adult males (17–58 years) and to be present in more than 50 % in men over 44 years of age [1]. In a group of hospitalized patients, ages 58 to 71 years, the mean prevalence was 65 %, and it increased with age and body mass index (BMI). With a BMI of 25 or greater, the prevalence was ~80 % [4]. Since the prevalence of palpable breast tissue (PBT) is so high, usually bilateral, and nearly always asymptomatic, the administration of a drug and the concurrent detection of gynecomastia could be purely coincidental, or more likely if pain/tenderness (mastodynia) is present the breast enlargement represents an independent (unique) syndrome, the etiology of which may or may not be related to a specific drug. This would particularly be the case if it is unilateral.

We have reviewed the English language literature from 1946 to January week 4, 2015 in an attempt to determine the types of data that frequently are included in reviews as indicative of drug-induced development of gynecomastia.

## Methods

Originally, the literature was searched in PubMed and the Ovid databases from the 1950s to April 2012 with the search terminology of “gynecomastia, drugs/medications.” Eighty-five publications were identified and were used in the review. Fourteen additional articles were found and added to the list for a total of 99. Seventy-six were case reports. Seven were series of HIV-infected patients treated with antiretroviral regimens. There were nine reports of population-based studies (prospective or retrospective). Subsequently, at the recommendation of one of the reviewers, the literature was search again using Ovid/Medline and PubMed for each of the 49 individual drugs listed in Table 1, crossed with the term gynecomastia. The date range of the search was 1946 to January 2015. From this search, we added 22 new cases, referred to in 9 additional references.

**Table 1:** Medications reported to be associated with the appearance of gynecomastia

	Drug	Reference
1	Amlodipine	[5 ^*^, 6]
2	Atorvastatin	[7]
3	Benserazide	[8]
4	Captopril	[9, 10]
5	Cimetidine	[11–15]
6	Cladribine	[16]
7	Combination, cytotoxic agents	[17–19 ^*^, 20–22]
8	Cyclosporine	[23 ^*^]
9	Dasatinib	[24]
10	Diazepam	[25–27]
11	Didanosine	[28]
12	Diethylproprion	[29 ^*^]
13	Digoxin	[30, 31]
14	Diltiazem	[32 ^*^, 33]
15	Domperidone	[34]
16	D-penicillamine	[35]
17	Etritinate	[36]
18	Efavirenz (HIV)	[37–39 ^*^]
19	Fenofibrate	[40]
20	Finasteride	[41 ^*^, 42 ^*^, 43 ^*^, 44, 45 ^*^, 46, 47]
21	Fluoresone	[48 ^*^]
22	Fluoxetine	[49, 50]
23	Gabapentin	[51]
24	HAART^^	[52–56 ^*^, 57–62]
25	Imatinib	[63 ^*^, 64]
26	Indinaver (HIV)	[65, 66 ^*^]
27	Isoniazid	[67–71]
28	Ketoconazole	[72 ^*^, 73]
29	Marinol	[74]
30	Methotrexate	[75, 76]
31	Metronidazole	[77]
32	Nettle	[78]
33	Nifedipine	[32, 79 ^*^]
34	Omeprazole	[80, 81]
35	Paroxetine	[82]
36	Phenytoin	[48 ^*^, 83, 84 ^*^]
37	Pregabalin	[85 ^*^]
38	Ranitidine	[86]
39	Rosuvastatin	[87, 88]
40	Saquinavir (HIV)	[89 ^*^, 90]
41	Spironolactone	[13, 29, 91–99]
42	Stavudine	[28, 100]
43	Sulindac	[101]
44	Sulperide	[102]
45	Sunitinib	[103]
46	Tandospirone	[104]
47	Thalidomide	[105, 106]
48	Theophylline	[107]
49	Venlafaxine	[108]
50	Verapamil	[32 ^*^, 13]
51	Vincristine	[109 ^*^]

* Indicates more than 1 case

^^ Highly Active Antiretroviral Therapy

Publications of an association with a drug no longer used, such as digitalis, and methyl-dopa, were not reviewed. We did not attempt an exhaustive review of the literature or of postmarketing reports of gynecomastia as an adverse effect of medications such as in the Food and Drug Administration (FDA-Medwatch) database. How gynecomastia is defined and ascertained is not readily available in these publications. Medications known to cause androgen deficiency, e.g., GnRH agonists and high-dose ketoconazole (600 mg/day or greater), were not included.

In this review, we attempted to determine (1) how the diagnosis was made; (2) the presence or absence of mastodynia; (3) whether the “gynecomastia” was unilateral or bilateral; (4) whether hormonal studies were done, i.e., testosterone/estradiol data; and (5) whether either the testosterone was low, the estradiol high, or the estradiol/testosterone ratio was abnormal. We also determined whether (6) a remission or disappearance of the mastodynia and/or breast “swelling” occurred over time and (7) whether a drug rechallenge test was done. Last, (8) ultrasound, (9) computed tomography (CT), and (10) histological data were recorded. It should be noted that with use of these procedures, a diagnosis of “gynecomastia” generally was made based merely by the presence or absence of breast tissue.

In addition, information available on the prevalence and characteristics of the reported gynecomastia in some population-based studies were reviewed.

The inclusion criteria were as follows: (1) any case report in which the data were sufficient for us to evaluate under the headings on Table 2, (2) Publications were in English, and (3) publications were available through the Veterans Administration or University of Minnesota library systems. The only exclusion criterion was references in which the data were insufficient for analysis.

**Table 2:** Analysis of case reports

Category	Yes^**^	No	Unknown	Total		Ratio	%
					Y + N	Yes/No	Yes/(Yes+No)
Pain/tenderness @ Dx *	103	30	26	159	133	3.4	77 %
Mastodynia ±	104	27	28	159	131	3.9	79 %
Unilateral	84	57	18	159	141	1.5	60 %
Hormones Checked	91	63	5	159	154	1.4	59 %
Hormones wnl ^	74	17		91	91	4.4	81 %
Remission	100	19	40	159	119	5.3	84 %
Rechallenge	11	148		159	159	0.07	7 %
Ultrasound	21	138		159		0.15	13 %
Mammography	46	113		159		0.41	29 %
Histology	22	137		159		0.16	14 %

* Presenting complaint by the patient

** Yes indicates that category was present and/or observed

± Noted by patient and/or the health care provider

^ Within normal limits

## Results

The medications reported to be associated with the appearance of gynecomastia are indicated in Table 1. There were 49 different medications implicated. In addition, some antineoplastic and antiretroviral regimens were reviewed. A total of 159 cases were reported and reviewed. There were 63 single case reports. More than one case was reported in 24 publications. There were 24 population-based studies of which 8 were highly active antiretroviral therapy (HAART)-treated patients with human immunodeficiency virus (HIV) infections.

The 49 single medications and the two combination regimens implicated in the genesis of gynecomastia and the references are indicated in Table 1.

### Analysis of case reports (Table 2)

#### Diagnosis of gynecomastia

How the diagnosis of “gynecomastia” was made was stated in 133 cases.

In 77 % of the cases, the patient experienced mastodynia and then noted a “swelling” of the breast. The patient commented on this to the provider who then initiated an evaluation. In the remaining cases, an increase in breast mass was mentioned by the patient or the provider. Most commonly, the patient mentioned an enlargement, and the provider merely confirmed it visually or by a superficial examination.

#### Mastodynia

The presence of pain or tenderness was reported in 104 cases (79 %). In 28 cases, a comment was not made regarding the presence or absence of mastodynia.

#### Unilateral gynecomastia

In the 141 cases in which it was determined, it was unilateral in 84 (60 %). In 18 cases, comments regarding this were lacking.

#### Hormonal profile

A hormonal profile (testosterone, estrogen concentrations) was reported in 91 cases. They were within the reference range in 74 (81 %).

#### Remission

In 119 cases where it was commented on, a remission occurred in 100 (84 %).

#### Rechallenge test

A rechallenge test was done in 11 of the case studies. In all 11, a recurrence of breast enlargement and/or mastodynia was reported after the drug was restarted. The drugs involved were nifedipine [32], sunitinib [103], isoniazid [67], fenofibrate [40], metronidazole [77], ranitidine [86], finesteride [41] (two subjects), methotrexate [110], omeprazole [111], and amlodipine [5]. A proposed mechanism in each case was not presented. In one report when the patient was restarted on metronidazole [77], he complained of bilateral breast tenderness within 3 weeks of restarting therapy, and the drug was stopped. Palpable breast tissue had not occurred.

#### Ultrasonography/mammography

Among the single case reports, “gynecomastia was confirmed” in 21 reports by ultrasonography (13 %). It was “confirmed” by mammography in 46 cases (29 %).

#### Histology

Histological data were obtained in 22 cases (14 %). Only a fine needle aspiration was done in 9 of these cases. Often, the objective was to rule out a malignancy.

In this regard, a retrospective histological evaluation for gynecomastia was published several years ago [112]. In a review of 351 patients with histologically proven gynecomastia, 335 of which were surgical specimens, there were 103 patients whose etiologies were considered to be idiopathic. Of these, the gynecomastia was unilateral in all but 10 (90 %). In 38 patients whose gynecomastia was suspected to be drug induced, 22 were bilateral (58 %). However, this included patients treated with hormones as well. Although not quantified, the majority of patients presented with pain or breast tenderness. The authors commented that idiopathic and “drug induced” gynecomastia tended to be more commonly unilateral whereas in those with a presumed hormonal etiology it was bilateral.

A florid-type histological appearance was more common in those who had gynecomastia for 4 months or less. Also, “mastitis” was observed nearly exclusively in those subjects categorized with florid-type histology [112].

### Population-based reports

Eight publications in which gynecomastia had been reported in patients with human immunodeficiency virus (HIV) infections receiving (highly active antiretroviral therapy) HAART treatment will be reviewed only briefly. The results are shown in Table 3. In general, the prevalence was either not indicated or was lower than expected in the general population. Also, whether the noted breast enlargement was new or not is sometimes difficult to determine. However, in the great majority, it was unilateral, frequently presented with pain/tenderness, and in general was not associated with a hormonal abnormality when this was determined. Thus, the characteristics of the reported gynecomastia were similar to those reported with a variety of other single medications. As with the single medications, the role of the HAART treatment, if any, in the genesis of “gynecomastia” is uncertain.

**Table 3:** Subjects with HIV

Study type	Total #	# w/ Gyneco-mastia	Method of diagnosis	Hormone profile
Population Paech et al. [57]	470	24 (17 unilateral)	Most visually.	Not done
Population L.Piroth et al. [58]	239	6	First observed by patients; confirmed by “clinical investigation.”	FSH, LH, TSH, cortisol within reference range. In 3 cases, testosterone low.
Population Mira et al. [59]	1304	30 (16 unilateral) 25 presented with pain	Diagnosis made if all the following were present: 1) Patient noted enlargement 2) Examination done 3) Ultrasound	Nested control comparison done (n = 30) bioavailable testosterone lower, free testosterone same as in those with gynecomastia.
Population Manfredi et al. [60]	492	14 (4 unilateral), 9 presented with tenderness/pain	Diagnosis made by patient. Observation followed by ultrasound.	Hormone data not reported. Stated that “Endocrine abnormalities were excluded”.
Population Strub et al. [61]	47	Breast pain/tenderness documented in 27 of 47. PBT present in only half	ND	Testosterone levels documented to be low in 4 subjects.
Population Allen et al. [113]	259	13 of 15 unilateral	Fine Needle Aspiration Cytology (FNAC)	ND
Population Garcia-Beneyas et al. [52]	1400	33 (31 unilateral, later developed bilateral)	Mastodynia	ND

*ND* not determined, *PBT* palpable breast tissue

Rechallenge not done in any

Of interest, in reference [52], spontaneous resolution occurred in 20 of 21 patients monitored for more than 1 year in spite of continuing the HAART regimen. Also, two cases have been reported in HIV-infected men that were not attributed to medications [53].

### Cohort studies analyzed by specific medication

#### Ketoconazole

There were several publications of an association of gynecomastia with ketoconazole. However, only two publications of the same three patients who met our criteria were found. In 40 patients being treated for a fungal infection with ketoconazole (200–400 mg/day), 3 complained of mastodynia, and bilateral gynecomastia was detected by physical examination after 3–6 weeks of treatment [72]. The examination technique was not specified. Hormonal studies were done in two patients and were within normal limits. A rechallenge was not done. Later, the same authors reported data on 60 patients, but there was no further increase in patients with gynecomastia [73].

#### Imatinib

In a carefully done prospective study of 38 men being treated with imatinib for chronic myeloid leukemia, breast mass changes were determined by direct sequential measurement [63]. Seven patients developed palpable breast tissue equal to or greater than 2 cm in diameter. The treatment itself tended to be associated with reduction in the free and total testosterone concentration in all of the subjects, but the decrease was in general greater in those who developed breast enlargement. Estradiol data apparently were obtained but not reported. The number reporting pain or tenderness was not indicated, but it was stated to have occurred often. Also, whether the breast enlargement was unilateral or bilateral was not stated. However, in at least one case, it was unilateral. A rechallenge was not done.

In a subsequent series of 44 patients being treated for a gastrointestinal stromal tumor, six developed a “painful lump under bilateral nipples” [64] which measured 2.5 to 5 cm. Testosterone was within the reference range, but the estradiol was modestly elevated in three patients. Over time, the “breast lesions” decreased in size in three and disappeared completely in two [64].

#### Cimetidine

There are several reports of an association of cimetidine with gynecomastia. Only three are reviewed here as typical of the data. In the first study [11], cimetidine, 1.6 gm daily, was administered to 25 patients with gastric symptoms. After treatment for 4–9 months, five patients reported soreness of one or both nipples, associated with breast swelling. How gynecomastia was confirmed was not indicated. Testosterone and estradiol were within the reference range. A rechallenge was not done.

In the second study [12], cimetidine was administered to 22 patients for “gastric hypersecretory states.” The typical dose was reported to be 3.5-fold (5.3 g daily) greater than a typical dose used to treat peptic ulcer disease. Nine complained of breast changes, 8 of which noted increased nipple sensitivity or breast pain. Gynecomastia was reported in 5. However, how the diagnosis was documented is unclear. Hormonal studies were not done. A rechallenge was not done.

The prevalence of gynecomastia also was reported in 81,535 males using data obtained from UK general practitioners, who agreed to provide data on patients being treated with proton pump inhibitors/antacids (cimetidine, misoprostol, omeprazole, or ranitidine) [13]. New onset gynecomastia was reported in 153. The gynecomastia was attributed exclusively to the use of cimetidine and was dose dependent. Information for 80 of these cases was available. In this group, 67 were self-reported, and 11 were incidental findings. Data were not available in two cases. Whether the patients presented with mastodynia was not stated. Thirty-four were unilateral, 38 were bilateral, and in 8, this information was lacking. In 37, there was complete regression, and it was partial in 14. Hormonal studies were not done. A rechallenge was not done. The gynecomastia was attributed to a cimetidine induced increase in estradiol due to impaired conversion to the inactive 2-hydroxy-estradiol product, as reported by Galbraith and Michnovicz [114]. However, as indicated above, hormone data were not obtained and based on the data obtained in reference [11], this explanation is unlikely.

Cimetidine is not commonly used at present, particularly in the doses used in the past. It is available over the counter in smaller dose sizes in the USA, and to our knowledge, an association of gynecomastia with cimetidine has not been reported in the last 10 or more years. This is as expected since in the postmarketing surveillance of 763 patients treated with cimetidine, the overall prevalence of “gynecomastia” was 0.2 % (quoted in reference [12]).

In summary, although cimetidine is rarely used in USA at the present time, data obtained in the past suggest that cimetidine in high doses can induce nipple tenderness, mastodynia, and transient breast swelling.

#### Omeprazole

In a retrospective review of cases of gynecomastia from the “Spanish Pharmacovigilance system” [80], 24 patients on treatment with omeprazole were identified as having gynecomastia in the 2007 year database. Here, the relative odds ratio for omeprazole exposure showed a statistically significant elevation in comparison to those with no exposure. However, how the gynecomastia was diagnosed and characterized was not reported.

#### Spironolactone

The presence or development of gynecomastia has been reported in several publications. Only representative reports are included here.

In a prospective study [91], 9 subjects received 400 mg of spironolactone for 24 weeks. Six developed gynecomastia. In none was the diameter more than 2 cm. In the same report, nine additional subjects received 100 mg for 4 weeks, then 400 mg for another 4 weeks. In none of these subjects did gynecomastia develop. Whether the breast enlargement regressed is unknown. One subject dropped out because of painful gynecomastia.

In the second prospective study [92], spironolactone or potassium canrenoate (a product of spironolactone metabolism) was administered to 44 subjects with liver cirrhosis and ascites. Fourteen received 100 mg/day of spironolactone and 30 received 100 mg/day of potassium canrenoate. All of those receiving spironolactone developed gynecomastia. Gynecomastia developed in 16 of the 30 who received potassium canrenoate. Here, gynecomastia was defined as a palpable, discrete button of firm subareolar tissue at least 2 cm in diameter. Whether the palpable breast tissue was new or associated with pain/tenderness cannot be determined from the publication. In this study, the testosterone, dialyzable testosterone, estradiol, LH, FSH did not change significantly.

In a case control study [93], 16 subjects were studied, 6 of whom received spironolactone and 10 were matched controls. All of those receiving spironolactone complained of breast swelling but apparently not pain/tenderness. All had been on 200–400 mg of spironolactone for 4 to 13 months before the study. Gynecomastia did not develop in the ten controls. The blood testosterone levels were significantly less and blood estradiol levels significantly greater in the spironolactone group, suggesting that spironolactone does alter peripheral metabolism of testosterone in some men and results in changes in the ratio of testosterone to estradiol. After withdrawal of the spironolactone, “the gynecomastia disappeared.”

It should be noted that the doses of spironolactone used in the above studies were greater than those commonly used clinically.

The presence or development of gynecomastia also has been reported retrospectively in several large studies. In the randomized aldosterone evaluation trial, self-reported gynecomastia occurred in 1 % of patients receiving a placebo but in 9 % receiving 25 mg of spironolactone [94]. In another retrospective study, the prevalence of gynecomastia was 13 % among 699 men treated for hypertension with spironolactone, the prevalence also was highly dose dependent. In those receiving greater than 150 mg/day, the prevalence was 52 %. Unfortunately, how the gynecomastia was detected and defined was not stated [95]. In another retrospective analysis of an open-label study, the reported prevalence of gynecomastia or breast discomfort was 6 % in patients when on doses of 25–50 mg/day [115]. Again, how gynecomastia was diagnosed was not stated.

Finally, in a retrospective study of 13,349 hospitalized medical patients monitored in a drug surveillance program, 788 of whom received spironolactone during 1 or more admissions, gynecomastia as an adverse reaction was reported in only 1.2 % [96]. In another retrospective study of 699 men receiving spironolactone alone or in association with another antihypertension treatment, gynecomastia was reported to occur in 13 % but was dose related [95].

The mechanism by which spironolactone induces modest gynecomastia remains unknown. It is speculated that spironolactone or a metabolic product of spironolactone [116] binds to the androgen receptors and displaces testosterone. Thus, spironolactone may act as a competitive inhibitor of androgen action. Indeed, it is used to reduce sexual hair growth in hirsute women with and without the polycystic ovary syndrome [117]. However, Stripp et al. [97] report that there is no plausible mechanism to explain spironolactone induced gynecomastia, as the drug has been shown to cause changes only in progesterone and 17-hydroxyprogesterone values.

### A treatment regimen for myeloma

In this retrospective study [20], during 1975–1979, nine patients diagnosed with myeloma were treated with a cyclophosphamide, prednisolone, and melphalan regimen. Four patients who developed gynecomastia were considered to be cases, whereas 5 who did not were considered to be controls. Gynecomastia was predominantly bilateral. How the gynecomastia was diagnosed was not indicated. An increase in free estradiol and a relative decrease in testosterone were only present in those with gynecomastia. Gynecomastia as a side effect of this regimen has not been reported since.

## Discussion

This review of the literature documents an extraordinary number and assortment of drugs reported to be associated with the diagnosis of gynecomastia. Most are only single case reports or small series. In the great majority of cases, the initial observation was by the patient and consisted of a painful or tender breast followed by a reported enlargement of the breast. Also, most commonly only a single breast was involved, although in some, it became bilateral later in the course. Also in nearly all cases, the pain/tenderness and the breast “swelling” resolved over time without specific treatment. Indeed, the breast enlargement was often described as disappearing completely. When determined, a hormonal etiology generally could not be documented, although speculated on frequently.

Ultrasound and/or computed tomography (CT) has been used to confirm the presence of gynecomastia. However, these data generally only indicate the presence or absence of detectible breast tissue. Whether it is new or not cannot be readily determined. The latter issue also is a limitation in interpreting breast histology. Although “florid” and “fibrotic” histological criteria have been used to estimate the acuity of breast enlargement [118], this has not been well documented. In addition, in some reports, an inflammatory component has been mentioned [112, 119, 120]. An increase in hyaluronic acid also is commented on, suggesting the presence of stromal edema [120].

As reviewed above, mastodynia with the subsequent observation of breast swelling has been reported to be associated with numerous medications. However, mastodynia associated with or without a documented acute enlargement of the breast occurs in men not receiving any medication.

For example, “idiopathic gynecomastia” was reported in 52 healthy men in the military [121]. All presented with breast tenderness and 15 complained of pain. In 41, breast enlargement was unilateral (79 %). A spontaneous remission in tenderness and breast size occurred over time. Thus, the “gynecomastia” in these men not receiving any medication was very similar to that reported to be associated with specific medications listed in Table 1.

As with the medication-associated reports, these signs and symptoms also suggest the presence of an acute syndrome and not the traditionally described chronic, asymptomatic gynecomastia. It also should be noted that in the above report [121], 1300 young military men requested surgical treatment for symptomatic gynecomastia over a 15-year period (an average of 87 cases/year).

Overall, current data suggest that for most drugs, their implication in the genesis of palpable breast tissue remains speculative. Although limited in scope, data implicating very high-dose cimetidine in the genesis are compelling [11, 12]. Imatinib also may induce breast enlargement, but the circumstances in which it is used complicate interpretation [63, 64]. Also, in a few case reports, a rechallenge test was done and was positive. In these reports, a cause and effect relationship probably was present. However, it likely represents an idiosyncratic response and was not hormonally mediated, since the medications involved have been widely used, but mastodynia and/or breast enlargement rarely been have reported. Again, the potential mechanism remains unknown.

Regardless of an unidentified mechanism, the high frequency of mastodynia and of single breast involvement associated with reports of drug-induced gynecomastia is impressive. These observations make it difficult to attribute their genesis to a circulating hormonally mediated mechanism, and indeed, in the majority of cases the hormone profile was within the respective reference ranges.

Spironolactone is an exception. That spironolactone administration can induce breast enlargement when ingested in high doses is well documented, and although the mechanism remains uncertain, it does appear to be hormonally mediated, i.e., an induced end result of an imbalance in the functionally mediated ratio of estradiol/testosterone. Again, the data are limited, but they suggest that the breast enlargement more commonly is bilateral and not frequently associated with the presence of pain and/or clinically significant tenderness, i.e., the data for spironolactone are more compatible with a hormonally mediated mechanism than that observed with other medications.

This also may be the case with the administration of cytotoxic agents for malignancies. Of a total of 21 cases associated with different medications and where the estradiol and/or the testosterone changes potentially could explain the onset of gynecomastia, cytotoxic agents were involved in 15. In addition, one was associated with finasteride where one might expect hormonal abnormalities.

Overall, we suggest that the general term of gynecomastia be replaced with more specific and descriptive terminology. Initially, we suggest that the term gynecomastia or preferably andromastia be divided into two large categories: acute with or without mastodynia and chronic.

## Conclusion

In conclusion, whether most of the medications implicated in the genesis of gynecomastia represent a true cause and effect relationship remains unknown. Most commonly, the syndrome presents as a unilateral onset of pain and/or tenderness followed by a reported enlargement of the breast. Generally, a hormonal etiology cannot be implicated. The symptoms and the breast enlargement spontaneously regress or disappear entirely, and no specific treatment or further evaluation is required. This includes ultrasound, mammogram, and biopsy. Generally, it can be treated symptomatically and not interfere with the continued use of the drug, if the latter is considered to be necessary.

The breast enlargement associated with the use of spironolactone is different. It is commonly bilateral, not associated with pain or tenderness, is dose dependent and is hormonally mediated, i.e., it likely represents an acute onset of traditional gynecomastia.
